# Microbiological contamination of cubicle curtains in an out-patient podiatry clinic

**DOI:** 10.1186/1757-1146-3-26

**Published:** 2010-11-18

**Authors:** Ria Woodland, Deborah Whitham, Bill O'Neil, Simon Otter

**Affiliations:** 1 School of Health Professions, University of Brighton, 49 Darley Rd, Eastbourne, East Sussex, BN20 7UR, UK; 2 Eastbourne District Hospital, East Sussex Hospitals NHS Trust, Kings Drive, Eastbourne East Sussex, BN21 2UD UK

## Abstract

**Background:**

Exposure to potential pathogens on contaminated healthcare garments and curtains can occur through direct or indirect contact. This study aimed to identify the microorganisms present on podiatry clinic curtains and measure the contamination pre and post a standard hospital laundry process.

**Method:**

Baseline swabs were taken to determine colony counts present on cubical curtains before laundering. Curtains were swabbed again immediately after, one and three weeks post laundering. Total colony counts were calculated and compared to baseline, with identification of micro-organisms.

**Results:**

Total colony counts increased very slightly by 3% immediately after laundry, which was not statistically significant, and declined significantly (p = 0.0002) by 56% one-week post laundry. Three weeks post laundry colony counts had increased by 16%; although clinically relevant, this was not statistically significant. The two most frequent microorganisms present throughout were *Coagulase Negative Staphylococcus *and *Micrococcus *species. Laundering was not completely effective, as both species demonstrated no significant change following laundry.

**Conclusion:**

This work suggests current laundry procedures may not be 100% effective in killing all microorganisms found on curtains, although a delayed decrease in total colony counts was evident. Cubicle curtains may act as a reservoir for microorganisms creating potential for cross contamination. This highlights the need for additional cleaning methods to decrease the risk of cross infection and the importance of maintaining good hand hygiene.

## Background

Exposure to pathogens on contaminated healthcare garments, uniforms, curtains and other fabrics can occur through direct contact or indirectly through airborne particle spread [1,2]. Infection control procedures play an important part in all clinical settings to prevent and reduce the rate of cross-infection. Scrupulous hand washing by healthcare staff before and after contact with patients and before any procedure is reportedly the single most important infection control measure [3]. However, there are various items that are touched after hand washing and prior to patient contact (e.g. clinical surfaces and/or cubicle curtains) that could be contaminated with microorganisms. Therefore, the potential for cross infection is increased with frequent contact with cubicle curtains [4]; particularly as some bacteria are able to survive on clinical fabrics for extended periods [5].

The podiatry clinic is unique in the nature of treatments involved, as a considerable amount of human proteins/tissue (mainly epidermis) can be deposited in the cubicle and dispersed into the surrounding environment. Curtains that surround the cubicle when drawn to provide patient privacy might disturb particles and microorganisms that could potentially increase the risk of airborne transmission and cross infection [4]. Curtains are widely used in acute units to provide privacy for in-patients who may be seen by several health care professions. Equally, in Community settings, particularly those with multi-chair clinics and possibly in private practices, curtains can serve a useful purpose. Healthy, intact skin serves as a formidable protective, however a significant proportion of people attending a podiatry clinic have diabetes or are immune-compromised. These populations are more susceptible to a wound and/or infection and it is of paramount importance to minimize the risk of cross infection [6-8]. At the time of the study cubicle curtains were cleaned by the local hospital laundry department where they were washed according to Health Service Guidance (HSG(95)18 - (65°C for not less than 10 minutes, or 71°C for not less than 3 minutes)). The aim of this study was to investigate the common microorganisms present on cubicle curtains in a podiatry clinic and establish the effectiveness of current cleaning strategies on the magnitude of colony counts.

## Methods

### Subjects and setting

Microbiological swabs were taken from 20 cubicles within clinics at a university-based, outpatient podiatry clinic. Each cubicle curtain was 249 cm long and 245 cm wide with a 12.5 cm distance from the ground. The curtains were made of 60% polyester and 40% cotton, drawn around an overhead track completely enclosing the treatment cubicle for patient privacy pre and post treatment. Ethical approval was granted from University of Brighton School of Health Professions research governance panel.

### Pilot studies

Swabbing the entire curtain area every time was clearly impractical. It would be possible, in theory, to swab a small square area and reconstitute in a known volume of fluid, however, this would still be unrepresentative of the whole curtain. During pilot observation work it was noted curtains were most commonly touched in the middle when being drawn or opened. Therefore it seemed practical, convenient and most importantly, clinically relevant to swab the central section of each curtain (measuring approximately 30 cm × 20 cm) at the edge, as opposed to the middle body of the curtain. A technique was piloted whereby three separate cubicle curtains were selected at random and swabbed where it was observed most likely to be touched when being used. All three curtains provided positive colony counts. As expected the number of colonies varied, (curtain A 18 colonies, curtain B 90 colonies and curtain C 32 colonies); however this approach indicated that the swabbing technique used (detailed below) was an effective method of determining both colony counts and enabled the identification of any micro-organisms present.

### Data collection

**Figure 1:**
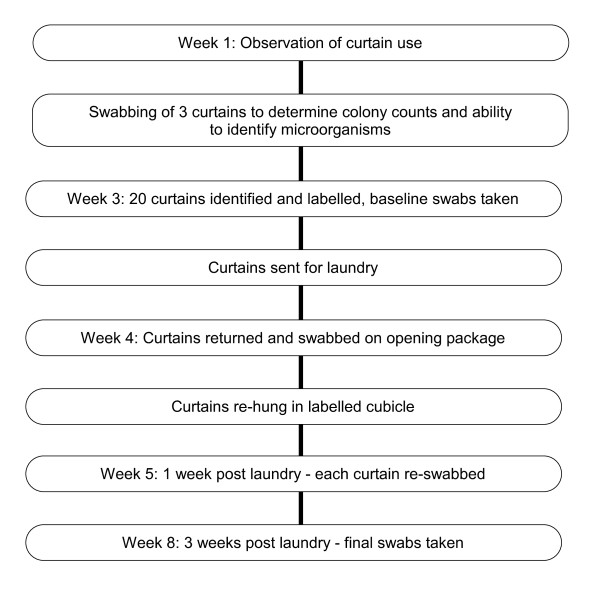
**Chronology of data collection**.

All microbial swabs were taken over a two-month period, the chronology of which is illustrated in Figure 1. For each swab sterile saline solution (0.9% sodium chloride) was poured into a sterile galley pot and placed on a sterile field. To assist with the collection of microorganisms each swab was moistened with the sterile saline solution. Additionally, sterile gloves were worn to prevent inadvertent contamination with skin commensals. To culture the microorganisms the swab was then spread over 7% horse blood agar plates supplied by the local Microbiology department.

A baseline swab was taken from 20 cubicles prior to the curtains being sent to the hospital laundry. Once cleaned, curtains were packed in plastic covering for return. The individual horse blood agar plates were labelled containing the cubicle number and directly sent to the microbiology laboratory. On return laundered curtains were removed from the plastic packaging, immediately swabbed and then placed back on to each of the numbered cubicles. A third group of swabs were taken using the technique described above, one week post laundry and then the final swabs collected three weeks post laundry.

### Culturing of samples

The 7% horse blood agar plates were processed and incubated aerobically at 37°C for 48 hours. Different colony types were counted and identified by Gram stain [9] and colony morphology [10-12]. Standard protocols were followed with regard to the identification process. Briefly, ammonium oxalate-crystal violet solution and Lugols iodine solution were applied for 30 seconds on each Gram stain then washed thoroughly with acetone-iodine decolouriser [13,14]. Additional, supplementary identification tests were carried out including DNase Test [15], Coagulase Test, growth on MRSA medium and Gram negative bacilli [16,17]. The DNase coagulase and growth on MRSA media to split staphylococci into three broad groups for identification - MRSA, MSSA and Coagulase negative staphylococci [18-20].

### Data analysis

The colony counts were recorded in separate tables for each time period and described using ordinal data. Formal hypothesis testing was not carried out as this study sought to determine quantitative and qualitative data pertaining to micro-organisms potentially present on cubicle curtains pre and post laundry. Previous work [4,5] has suggested that laundry procedures may not be 100% effective, but this type of work has not been carried out to this extent in an out-patient community podiatry setting. Data were not normally distributed either between curtains or on the same curtain pre and post laundry; therefore non-parametric statistical tests were employed. The Mann Whitney U-test was used to identify any statistically significant differences in colony counts from baseline. Computer assisted analyses of the colony counts were performed using the MINITAB (Pennsylvania USA) software package (version 14).

## Results

### Colony counts

Baseline swabs were taken in order to determine the quantity and class of microorganisms present before laundering. The total colony counts were then calculated and compared between baseline immediately after laundry as well as one and three weeks post laundry (table 1). At baseline colony counts totalled 1358. Immediately following laundry there was a small (3%) increase in colony counts from 1358 by to 1399 (this was not statistically significant (p = 0.96)). Comparing the colony counts from immediately after laundry to one week post laundry, colony counts decreased significantly by 56.4% from 1399 to 610 (p = 0.0002). The total count one week post laundry compared with three weeks post laundry revealed an increase of 16.4% from 610 to 710, while clinically meaningful over a short time period, these results did not reach statistical significance (p = 0.2).

**Table 1:** Total colony counts.

Timeframe	Colony count	% difference from baseline (p value)
Baseline	1,358	-
Immediately post laundry	1,399	+ 3.0 (p = 0.96)
One week post laundry	610	- 56.4 (p = 0.0002)
Three weeks post laundry	710	+16.4 (p = 0.2)

### Identification and analysis of microbiological species

A wide range of microorganisms were identified during the course of this study (table 2). The laundry process was not immediately effective on the *Micrococcus sp*., which remained fairly constant showing little change as a result of laundry (colony count 345 at baseline and 335 immediately post-laundry, declining to 250 three weeks post laundry). Numbers of *Bacillus Sp*. were fairly constant throughout the study (albeit in lower numbers than *Micrococcus sp*) signifying laundry had little or no effect in decreasing the colony counts (baseline colony count 20, rising to 25 immediately post-laundry and 22 three weeks post laundry). Laundry was found to be particularly effective against *Diptheroid *with a baseline colony count of 41 declining to 2 immediately post-laundry. Colony counts of *Coagulase negative Staphylcoccus *were the highest overall (950 at baseline) and these increased slightly to 1010 immediately post-laundry. Colony counts then fell dramatically to 150 one week post laundry, but had doubled to 300 by three weeks post-laundry. *Alpha-haemolytic Streptococcus *numbers were 0 pre-laundry to 1 immediately post-laundry. However, colony counts for this microorganism rose to 23 and 30 one and three weeks post-laundry respectively. Finally, while no colonies of *Staphylcoccus Aureus *were noted at baseline, there were 12 immediately post-laundry. Although colony counts had returned to zero by one and three weeks post-laundry.

**Table 2:** Identification of microorganisms.

	Timeframe			
**Microorganism**	**Baseline**	**Immediately** **post laundry**	**1 week post** **laundry**	**3 weeks post** **laundry**

Micrococcus Species	345	335	350	250
Coagulase Negative Staphylcoccus	950	1010	130	300
Environmental Gram- Negative Bacilli	8	30	45	50
Bacillus Species	20	25	40	22
Diptheroid	41	2	31	55
Esherichia Coli	0	2	0	0
Staphylcoccus Aureus	0	12	0	0
Alpha-haemolytic Streptococcus	0	1	23	40

## Discussion

This study has demonstrated the presence of a variety of bacteria on podiatry clinic curtains prior to and following an approved laundry process. Firstly, laundry was noted not to be 100% effective against all organisms. Additionally, during the 3 weeks post-laundry the number of colonies of microorganisms had started to rise: although this rise was not statistically significant our contention is that this is highly relevant clinically given the frequency with which curtains may be touched during clinical sessions. These findings are of particular significance as many of the organisms identified could cause potentially serious infections, especially in those patients who are immunosuppressed. The total colony counts measured in this investigation somewhat unpredictably demonstrated a small increase after washing, potentially suggesting that the cleaning procedure used was not completely effective in reducing the microbial load. Other explanations for this discovery could be that the curtains were contaminated whilst being handled at the laundry department, or that curtains were contaminated by other fabrics [8] during laundry. Previous work [21] reported that prior to laundry hospital linen was heavily contaminated with *Bacillus Cereus *and varying numbers of other micro-organisms, in particular, *Gram-negative Bacilli*, *Coagulase Negative Staphylococci *and *Bacillus Species*. Equally, the podiatry curtains could have been contaminated from previous washing of hospital linen within the Continuous Batch Tunnel Washer. It is not possible to know if curtains were cross contaminated by other linen, but this is a possibility. Moreover, this highlights the importance of appropriate hand decontamination following contact with curtains and prior to contact with patients. Additionally, previous work has speculated that bacterial spores can survive thermal disinfection, since not all parts of the machine may reach high temperatures throughout a day of laundering [22]. At the time of our study Department of Health (DoH) guidance was that 'all compartments of the Continuous Batch Tunnel Washer must be emptied at the end of each working day' [23]. However, others have questioned whether all laundry departments are able to fully adhere to DoH guidance due to the practicalities associated with continued use, restricted time and limited funding [5,7,21].

Previously, it has been highlighted that current disinfectant procedures are becoming increasingly ineffective in eliminating potential pathogens such as *Staphylococcus Aureus *[24]. A particularly disquieting finding from the current study revealed that curtains became contaminated with *S. aureus *after laundry, yet no presence of *S. aureus *was found from the baseline swabs (table 2). *S. aureus *typically forms part of the normal flora, living permanently on the skin surface [25], but can cause opportunistic infections. The increase in microbiological load noted between one and three weeks post laundry and the survival of opportunistic pathogens such as *S. aureus*, highlights the need for adherence to all decontamination procedures to reduce the risk of cross infection [26] particularly as the transmission of micro-organisms from the clinical environment to an individual is still possible [4,6,8]. For example, *Coagulase Negative Staphylococcus *is able to survive on various materials including plastic [5,8,27]. Our study did not find evidence of resistant bacteria such as *Meticillin-Resistant Staphylococcus Aureus *(MRSA) contaminating curtains, although previous work has reported that MRSA can survive longer on fabrics such as those used for curtains [28]. While effective cleaning can reduce the prevalence of MRSA in the clinical environment, there has been a continual increase in bacterial resistance [24]. Gram-positive bacteria are considered to be more sensitive to disinfectants than Gram-negative bacteria due to the composition of the cell wall [29] and differing resistance mechanisms [7]. Bacterial resistance to biocides could potentially be combined with resistance to antibiotics and has led microbiologists to express the need to establish the underlying mechanisms of resistance [30] to enhance effectiveness of current decontamination procedures [21]. Currently therefore, care must be taken when choosing from the wide range of cleaning products available to evaluate their activity against key pathogens [31].

The findings of this study do need to be seen in the context of some limitations. While the swabbing technique and site of the curtains was deemed sufficient in collecting pathogens, practicalities dictated that the size of the potential bacterial reservoir tested was only a proportion of the entire surface area of each curtain. Equally, it was beyond the scope of this study to determine if garments from different wards that could been highly contaminated were not separated prior to laundry to prevent further transmission of pathogens. An extension of this study could assemble valuable data in measuring the microbial load over a longer period of time to analyse and evaluate specific pathogens for growth and survival rates. A follow-up study under controlled conditions could time map the colonisation on clean, virgin curtains as opposed to those laundered and returned to the clinic. Finally, patterns of curtain usage could yield valuable data regarding potential cross-infection risks, particularly if a more frequent cleaning programme for the cubicle curtains to strengthen infection control is required. The use of newer techniques such as biofilm inhibitors could reduce microorganism growth rates as could the use of novel fabrics (e.g. silver impregnated) [32]. In the light of our study the use of disposable curtains that are regularly replaced would be recommended, but costs for any of these alternatives could be prohibitive depending on local circumstances.

In conclusion, the measurement of microorganisms on podiatry cubicle curtains found elevated colony counts of common pathogens risking the potential for cross-infection. This highlights the importance of existing cross-infection control measures such as effective hand washing. A newly devised cleaning programme for clinical curtains may be required to reduce the risk factors of a reservoir for infection and enhanced potential for bacterial resistance.

## Competing interests

The authors declare that they have no competing interests.

## Authors' contributions

RW conceived the study, collected data and performed data analysis. DW participated in study design and supervised data collection and analysis. WO participated in study design, analysed samples and identified microorganisms. SO assisted with data analysis, coordinated manuscript writing and submission. All authors read and approved the final manuscript.
